# Magnetic Hygiene

**Published:** 1888-12

**Authors:** S. Helen Clarke


### MAGNETIC HYGIENE.

### PART II.

Our life is divided in two distinct states : the wakeful state, which is one of activity, of labor, and the state of slumber, which is one of repose and quiet.

During the wakeful hours we expend a large amount of magnetic power, and that in proportion to the quantity and to the quality of the labor we perform. Much more of the power is consumed by mental exertion, or by occupation necessitating a constant employ of the intellectual faculties, than by the unconscious bodily functions, simple effects of will without thought, or by mechanical or machine-like labor, which is effected much in the same manner.

During sleep, when the intellectual faculties are comparatively at rest, this expenditure of magnetic force to the outward is reduced to its minimum, and more of it is absorbed in the building up of tissues and general strengthening of the physical organism.

We, therefore, understand how important to the maintenance and promotion of health is, first, a relatively moderate expense of our magnetic or vital strength, and how when compelled by circumstances or conditions to a large expenditure of it, we should take advantage of all the means provided by nature to supply the increased demand ; and second, to obtain a reasonable amount of uninterrupted sleep, and that under conditions that will not interfere with, nor lessen the full supply of magnetic force needed by nature in the process of reconstructing and strengthening that which has become impaired and weakened by labor.

Light, heat, the air we breathe and the food we absorb, are the natural avenues through which magnetic power is transmitted to the organism.

The sun, undoubtedly the great magnetic centre of our planetary system, is, through the genial influence of its vivifying rays, constantly calling into action the life principle and sustaining it in its numberless expressions.

Great benefit may be derived by moderate exercise in the sunlight at times when the heat evolved from it is bearable and pleasant ; also by exposing the body, or part of it lightly covered, to the influence of the sun's rays, until a gentle glow is felt and circulation is strongly attracted to the surface.

It is needless to say that in such instances the head should be sufficiently shaded, so as to bring no discomfort to the individual.

Sunlight and heat should also be allowed to freely penetrate our dwellings, especially our sleeping apartments. That, together with a thorough ventilation, will be found all powerful in expelling morbid conditions.

Although the food we partake of should be so prepared as to be easily masticated and digested, we should bear in mind that the process of cooking decomposes in whole or in part, the substances submitted to it, eliminating thereby the magnetism with which nature has permeated them, and which they retain to a considerable extent as long as their cellular organization is not interfered with.

Our diet to be hygienic, from a magnetic standpoint, should therefore consist in part at least, of such articles of food as may be partaken of in a raw state.

Milk, eggs, many of the vegetables, well ripened fruits and nuts, even some meats used in their natural state will be found of easier digestion and assimilation when properly masticated, and will be much better adapted to promote the vital energies of the organism.

Thus, as human magnets, we can draw from nature a supply of crude magnetic force sufficient to permit even a large expenditure of it during the wakeful state, provided that in the state of repose sleep may be obtained without a further depletion of our life's forces.

This, as well as the adaptation of animal and spiritual magnetism to our subject, we will consider in part 3rd. S. HELEN CLARKE.

